# 

**Published:** 1825-10-01

**Authors:** 


Bibliographical Record .. .. .. .. .. .. .. .. .. 591

### XIII.

### EXTRA-LIMITES.

I. Dr. Gordon Smith's Reclamation .. .. .. .. .. .. 596

II. Dr. Davis on the Forceps .. .. .. .. .. .. 597

III. General Meeting of the Associated Apothecaries .. .. .. 598

### XIV.

### INTELLIGENCE, CORRESPONDENCE, &c.

Anatomie Vivante, or Skeleton Importation Company.. .. .. 599

Dr. Heiniker.. .. .. .. .. .. .. .. .. .. .. 600

Sea Water Baths .. .. .. .. .. .. .. .. .. .. 601

Additional Subscribers .. .. .. .. .. .. .. .. 602

---

*Dr. Nuttall's Lectures.*

Sketch of the Arrangement of Diseases, as abbreviated from Dr. Nuttall's large Chart adopted in his Lectures.

---

### TO CORRESPONDENTS.

The Parallel is received from S. by the Foreign Post. It came on the 17th of September, after the Journal was closed. It will appear in our next.

Justus will see the subject he alludes to near the end of the Periscope in this Number.

The very valuable specimen of Diseased Bladder has been received from Dr. D. It is forwarded to the Museum of the College of Surgeons as a very rare and important Specimen.

### XII.

### BIBLIOGRAPHICAL RECORD;

OR,

*Works received for Review between the 15th of June, and the 15th of September, 1825.*

1. A Treatise on the different Methods of investigating the Diseases of the Chest, particularly Percussion, and the Use of the Stethoscope. Translated from the French of M. Collin, with an additional Preface. By W. N. Ryland, M.D. Small Octavo, pp. 67, with a Plate. Burgess and Hill, July, 1825.

☞ *This is a Translation of the little Work noticed at page 91 of Vol. I. of this Series. It is a very useful Pocket Companion while studying the Use of the Stethoscope, and the general Means of ascertaining the Nature of Thoracic Diseases.*

---

2. Observations on Gout, critical and pathological; or an Analytical Survey of the Views at present entertained of the Nature of that Disorder; with Practical Remarks on the injurious Effects of Colchicum, and on certain Modes of Diet. By A. Rennie, Surgeon, &c. 8vo. pp. 179. Underwoods, July 1825.

☞ *As the Author is preparing for the Press "a Treatise on Gout, pathological, therapeutical, and practical," we shall wait its appearance before we proceed to a Review of the present Work, which is only preliminary.*

---

3. Practical Commentaries on the present Knowledge and Treatment of Syphilis: with coloured Illustrations of some ordinary Forms of that Disease. By Richard Wellbank, Member of the Royal College of Surgeons, &c. 8vo. pp. 50. Longman's, 1825.

---

4. The Edinburgh Medical and Surgical Journal, for July 1825. 8vo. pp. 224, with plates. (*In exchange*).

---

5. Military Medical Reports; containing Pathological and Practical Observations, illustrating the Diseases of Warm Climates. By James M'Cabe, M.D. &c. 8vo. pp. 253. London and Cheltenham, 1825.

---

6. A Short Account of the celebrated Saratoga Mineral Water, in the State of New York, North America. 8vo. sewed, pp. 8. 1825.

---

7. Practical Remarks upon Indigestion; particularly as connected with Bilious and Nervous Affections of the Head and other Parts; including Observations upon Disorders and Diseases of the Stomach, and superior parts of the Alimentary Canal. Illustrated by Cases. By John Howship, Assistant Surgeon to the St. George's Infirmary, &c. &c. &c. 8vo. pp. 192. Longman's, July 1825.

---

8. A System of Anatomical Plates, with descriptive Letter-press. By John Lizars, F. R. S. E. Part VIII. The Brain, second and concluding Portion. Coloured after Nature. Price £1. 1s. coloured.

☞ *It is enough to say that this Portion is equal to any preceding one, and superior to several. The character of the Work is now established at home and abroad.*

---

9. A Course of Dissections for the Use of Students. By Herbert Mayo, Surgeon and Lecturer on Anatomy. 8vo. pp. 284, with Plates. Price 7*s* 6*d*. London, July 1825.

☞ *We have looked over this Course of Dissections, and we venture to predict that it will be found more completely adapted to the practice of the Dissecting Room than any Manual hitherto published in this country. It would be the better for an Index, in addition to the Table of Contents.*

---

10. The Medical Recorder of Medicine and Surgery. Nos. 25-6-7-8-9, for the years 1824-5. Conducted by Samuel Colhoun, M.D. &c. &c. Philadelphia, 1824-5. (*In exchange.*)

---

11. Oratio in Collegii Regalis Medicorum Londinensis Ædibus Novis, habita die Dedicationis Junii XXV, MDCCCXXV. Ab Henrico Halford Baronetto, Medico Regis Ordinario, Preside. Londini, 1825. Quarto, pp. 16.

---

12. Lofrede op Hermannus Boerhaave. Door J. L. Kesteloot, Aan Wien de Uitgeloofde gouden &c. &c. Leiden, 1825, pp. 75.

☞ *This Historical "Eloge" of the immortal Boerhaave procured for its able Author, Dr. Kesteloot of Ghent, the Grand Medal from the Academy of Amsterdam.*

---

13. Josephi L. B. de Quarin, Animadversiones Practicæ in diversos Morbos. 1. Morbi Acuti. 2. Morbi Chronici. Editio Viennensi, accatior atque emendatior. Curavit, præfationemque adjecit J. L. Kesteloot, M D. &c. Two volumes, 8vo. pp. 286 et pp. 253. Gand, 1820.

☞ *This Work is written in very perspicuous Latin, and contains a fair Epitome of the Nature, Symptoms, Causes, and Treatment of Acute and Chronic Diseases. Quarin was a Physician of considerable eminence at Vienna, about the middle of the last century. He was Physician to Joseph the Second, Emperor of Germany, who made him a Baron, and gave him a Thousand Golden Sovereigns for candidly telling his august Master that he had but a very short time to live. "Augustissimus Œges cordato Medico gratias quam maximas persolvit. Missis postridie ad eum litteris Autographis, quibus Baronum ordini adscribebatur* Quarinus, *titulisque hisce honorificentissimis insuper donum junctum erat mille nummorum imperialium Aureorum." We beg to return Dr. Kesteloot many thanks for the above volumes, and for the flattering expressions contained in the Letter which accompanied them.*

---

14 Remarks on Irritative Fever, commonly called the Dock-yard Disease; with Mr. Dryden's detailed Account of the Fatal Cases, including that of the lamented Surgeon, Dr. Bell. By John Butter, M.D. F. R. S. F. L. S. &c. Fellow of the Royal College of Physicians of Edinburgh, &c. &c. 8vo. pp. 302. Plymouth and London, 1825.

---

15. Note sur les Contractions Musculaires produites par le Contact d'un Corps solide avec les Nerfs, sans Arc Galvanique. Par W. F. Edwards, M.D. 8vo. sewed (Extract des Annales des Sciences Naturales) Paris 1825.

16. A Compendious System of Midwifery, chiefly designed to facilitate the Inquiries of those who may be pursuing this Branch of Study. Illustrated by Cases, with Fourteen Engravings. By WM. P. DEWEES, M.D. Lecturer on Midwifery, &c. 8vo. pp. 628. London (reprinted) 1825.

☞ *Dr. Dewees has, we believe, been a distinguished Practitioner and Teacher of Midwifery for* 30 *years past, in Philadelphia. His Work is greatly esteemed in his own Country, and we look upon it as one of the best systems of Obstetric Practice which we possess. We shall take an early opportunity of giving some farther account of the Work.*

---

17. Practical Observations on certain Pathological Relations which exist between the Kidneys and other Organs of the Human Body, especially the Brain, Mucous Membranes, and Liver. By JOHN FOSBROKE, Surgeon, Cheltenham. 8vo. pp. 144. Cheltenham and London, August, 1825.

☞ *In our next.*

---

18. Medical Researches on the Effects of IODINE in Bronchocele, Paralysis, Chorea, Scrofula, Fistula Lachrymalis, Deafness, Dysphagia, White Swelling, and Distortions of the Spine. By ALEXANDER MANSON, M.D. Physician to the General Hospital, and St. Mary's Hospital and Dispensary, Nottingham. 8vo. pp. 450. Longman's and Co. London, August 1825.

☞ *This is an important Work, full of facts, and sparing of theories We seriously recommend it to the attention of every practitioner, on account of the efficacy of the remedy, and the obduracy of the diseases in which it has been fully employed. More in our next.*

---

19. The Philadelphia Journal of the Medical and Physical Sciences, Edited by Drs. Chapman, Dewees, and Godman. No. 1 of a New Series. 1825. *(In exchange.)*

---

20. The Science of Surgery; or the Principles of Pathology made the Basis of Medical and Surgical Practice; by which the Healing Art is considerably simplified—established upon three Morbid Conditions of the System; and extricated from a Labyrinth of useless and erroneous Terms, Classes, Orders, &c. By W. W. SLEIGH, Esq. Lecturer in London on Anatomy, Physiology, and Surgery, &c. &c. Vol. I. 8vo. pp. 318. Anderson, London, August 1825.

---

21. The Syphonic Theory, or brief Observations on the Circulation of the Blood, and on Respiration as connected therewith. By EDWARD HOPLEY, Surgeon, R.N. 8vo. stitched, pp. 40. Price 1s. London, August 1825.

---

22. Conversations on the Theory and Practice of Physiological Medicine; or Dialogues between a Savant and a young Physician, a Disciple of Professor Broussais; containing a concise Exposition of the new Medical Doctrine, and a Refutation of Objections brought forward against it. Translated from the French. 8vo. pp. 326. August, 1825. Price 9s. bds

☞ *An amusing and very complete Exposition of the Doctrine of Broussais, commonly called the "New Physiological Doctrine," is now published in an English dress—and although we are far from agreeing with its celebrated Author in all his Doctrines, we think this Book is extremely well worth the perusal, and we accordingly have no hesitation in recommending it to all Book Societies, and to all individuals, concerned in the controversy now raging with such violence on the Continent.*

23. Elements of the Theory and Practice of Physic, designed for the Use of Students. By George Gregory, M.D. of the Royal College of Physicians of London—Secretary to the Medical and Chirurgical Society—Physician to the Small-pox Hospital, &c. &c. Second Edition, with Additions and Amendments. One volume, 8vo. pp. 670. London, August 1825. Price 16*s*. boards.

☞ *We had occasion to speak favourably of the first edition of the first volume. The two volumes, in this edition, are compressed into one, with very great improvements and considerable additions. The Work has been favourably received by the Public, and will unquestionably take its rank as a Class Book of no mean merit for the rising generation. We hope to notice the second Part (formerly the second Volume) on* Chronic Diseases, *in our next Number.*

---

24. Traité Anatomico-Pathologique des Fièvres Intermittentes, Simples, et Perniceiuses, fondé sur des Observations Cliniques, sur des faits de Physiologie et de Pathologie comparées, sur des Autopsies Cadavériques, et sur des Recherches Statistiques, recueillis en Italie, et principalement a l'Hopital de Saint Esprit de Rome, pendant les Années 1820-1-2. Par E. M. Bailly, de Blois, M.D. &c. &c. 8vo. pp. 600. Paris, 1825.

---

25. Tentamen Medicum Inaugurale de Usu Arsenici Medicinale. Auctore Jacobo Nash, M.D. Edinburgi, 1825.

☞ *A very creditable proof of having gained the summi Honores in the noble Faculty of Medicine.*

---

26. Disputatio Medica Inauguralis Quædam de Turberculis, Vomicis, et Hepatizatione Pulmonum; deque Cælo hæc ipsa excitante, complectens. By John Hammett, M.D. Surgeon in the Royal Navy, &c.

☞ *Dr. Hammett has not visited various parts of the globe as an inattentive observer. He has made numerous interesting remarks on the Medical Topography of different Countries and Climates, and on the prolific Causes which generate or excite into action those Tubercular growths in the Organ of Respiration which carry so many to an untimely grave. At the end of the Thesis is a letter from his venerable and veteran Preceptor, Dr. George Pearson—on the subject of Tubercles and Hepatization, containing the results of that distinguished Physician's long experience in these Complaints.*

---

27. A Synopsis, exhibiting at One View the probable Results, ex ordine, of the various Chemical Processes contained in the Pharmacopœia Londinensis, 1824, together with Test Questions of the Pupil's Knowledge of the same. By G. F. Collier. In a Sheet. August, 1825.

---

28. Disputatio Chemico-Medica Inauguralis de Sanguine Ægrorum. Auctor, Johannes Whiting, M D. Edinburgi, 1816.

☞ *In this Experimental Thesis Eight Tables are given, shewing the appreciable Differences between the blood of people in Health and of those in various states of disease, particularly as regards the relative proportions of serum, fibrin, &c. as also the comparative specific gravity, &c. In this branch of Medical Science, we mean Animal Chemistry, a wide field is open for talent, industry, and opportunity. We hope that at this moment there are active labourers in the vineyard.*

29. Lettres à un Medecin de Province; ou Exposition Critique de la Doctrine Medicale de M. Broussais. Par A. MIGUEL, M.D. 8vo. pp. 52. Paris, 1825.

☞ *M. Miguel is the ingenious and keen Critic who conducts the* GAZETTE DE SANTÉ *of Paris, and has, in this Volume, exercised his critical powers to the no small detriment of the "Physiological Doctrine." As we have given our sentiments on this doctrine in the first article of this Number, we need not repeat them here. We return M. Miguel our best thanks for this amusing and intelligent Critique.*

---

30. An Inquiry into the Seat and Nature of Fever, as deducible from the Phenomena, Causes, and Consequences of the Disease, the Effects of Remedies, and the Appearances on Dissection. By HENRY CLUTTERBUCK, M.D. Member of the Royal College of Physicians of London, and Senior Physician to the General Dispensary, &c. 8vo. pp. 494. (Second Edition) Anderson, September, 1825.

---

31. A List of Drugs and Chemicals, including the New Medicines, Horse and Cattle Medicines, Perfumery, and other Articles generally sold by Chemists and Druggists, arranged alphabetically under their English Names, with the Latin Synonymes in general Use; and also the altered Names in the New Pharmacopœia: to which are added the Doses. Intended as a Price Book. By a Chemist and Druggist. 8vo. pp. 67. Price 2*s.* 6*d.* Anderson, September, 1825.

---

32. The Works of Matthew Baillie, M.D. to which is prefixed an Account of his Life, collected from authentic sources. By JAMES WARDROP, Surgeon Extraordinary to the King, &c. &c. Two volumes, 8vo. pp. 257—and pp 407. Price 25*s.* boards. Longman and Co. September, 1825.

☞ *This Work contains all Dr. Baillie's scattered Writings, a few posthumous Papers, and his great Work on Morbid Anatomy. We shall notice it in our next.*

---

33. Gulielmi Harveii Exercitationes de Motu Cordis et Sanguinis; quas Notis pauculis instruendas curavit THOMAS HINGSTON, M.D. Societatis Regiæ Medicæ Edinburgensis Socius, nunc ex Collegio Reginæ Cantabrigiensi. Edinburgi, 1824.

☞ *Some Notice of this Edition in our next.*

---

34. A short Enquiry into the Capillary Circulation of the Blood; with a comparative View of the more intimate Nature of Inflammation: and an introductory Essay. By JAMES BLACK, M.D. S.R.N. Member of the Royal College of Physicians. Octavo, pp. 176. London, September, 1825.

☞ *This is an ingenious little Essay, which we shall take an opportunity of noticing hereafter.*

---

35. The New Medico-Chirurgical Pharmacopœia, being a Selection of modern Formulæ from the private and hospital Practice of the most eminent Members of the Profession in Europe and America. For the Use of Surgeons and Surgeon-Apothecaries. Third Edition, with considerable Additions. By a Graduate of a Scotch University, Member of the College of Surgeons, of London, &c. &c. Octavo, pp. 180. London.

☞ *It appears that this useful Compilation has gone through two large editions in a small space of time. "The selection of the Formulæ of this new*

*edition, and the remarks on them, were made by a general practitioner, who has been actively engaged in practice, and in accumulating facts, for thirty-five years; and he flatters himself that the additions the Work has received, will, in a practical point of view, be deemed, by competent judges, very valuable."* *This is a strong recommendation, and, indeed, the Work appears to be well calculated for the general Practitioner.*

---

36. On the Removal of Stone from the Bladder, without the Use of Cutting Instruments; containing a general review of the subject, together with a description and plates of the instruments invented by Dr. Civiale and others, and details as to the mode of using them. By H. G. Belinaye, Esq.

"On y trouve, parmi une foule de procedés que l'opinion ensevelit en naissant dans les livres de leurs auteurs, quelques procedés heureux que le genie enfanta, que l'experience a consacré."—*Desault, Operation de la Taille.*

London, Published by Callow and Wilson, Medical Booksellers, 16, Princes-street, Soho. MDCCCXXV.

---

37. Dissertatio Medica Inauguralis de Febre Bataviæ Endemica. Auctore Roberto Armstrong, M.D. et in Classi Britann. Chirurg.

☞ *This able Dissertation is full of valuable practical Facts drawn from Personal observation. It is very creditable to the Author.*

---

*** Note.—Authors and publishers will readily appreciate the importance of having their works recorded, with full length titles, on this list, which stands as a perpetual advertisement so long as the Journal lasts, and as far as it extends. The republication of the Journal in America enhances the advantages of the Bibliographical Record, on which no work can be entered, unless transmitted, free of expence, to the Editor, under sealed cover to the publishers, or in any other way most convenient to the parties concerned.

*N. B.—As the Bibliographical Record closes on the 15th of the month preceding publication, all Works received after that date necessarily stand over till next quarter.*

---

### XIII.

### *Additional Subscribers since last Quarter.*

Bech, H. Esq. Fellow of the Royal College of Surgeons, Needham Market, Suffolk.
Blundell, Dr. James, St. Saviour's Southwark.
Brabant, Dr. Physician, Devizes.
Coleman, William, Esq. to the East Indies.
Church, Mr. J. P. Newcastle.
Down, Dr. John Summers, resident Physician, Florence.
Drew, Mr. C. W. Student, Edinburgh.
Ducket, Mr. Newcastle.
Dwyer, Mr. Francis, Member of the Royal College of Surgeons of London, to the East Indies.
Edwards, Dr. W. F. resident Physician, Paris.
Fraizer, A. A.M. M.D.
Fulton, Henry, M.D. Leamington.
Greenhow, Mr. T. M. Newcastle.
Hewett, Dr. Fellow of the Royal College of Physicians of London, and Physician to St. George's Hospital, Albany.
Hughes, Mr. Surgeon, Suffolk.
Infirmary Medical Library, Newcastle.
Kay, Mr. Sen. Hon. East India Company's Service, Madras.
Kestleloot, J. L. Medicinæ, Chirurgiæ et Artis Obstetricæ Doctor; Professeur de Medecine à l'Université Royale de Gand, &c. &c.
Kellatt, Richard, Esq. Madras.
Keenlyside, Mr. Richard, Newcastle.
King, Dr. B. W. Glasgow.
Lambden, Mr. Surgeon, Lincolnshire.
Mackintyre, Mr. Newcastle.
Marsh, Mr. John, Newcastle.
Medical Society of Newcastle-upon-Tyne.
Mills, Mr. Nicholas, to Bogota.
Mitchell, Mr. Surgeon, Leadenhall-street.
Ormond, Mr. Surgeon, Hon. Comp. Service, Surat, East Indies.
Parkins, Mr. Henry, Surgeon, Barking.
Price, Mr. William, Weston-super-Mare, Somersetshire.
Rackster, John, Esq. Surgeon, Pershine.
Scott, Mr. Jos. Hemphill, Surgeon of the Royal Tyrone Staff, Member of the Royal College of Surgeons of London, Caledon, Ireland.
Sillery, Robert, M. D. Member of the Royal College of Surgeons, and Assistant Surgeon to the Forces.
Sleigh, Mr. W. W. Member of the Royal College of Surgeons, and Lecturer on Anatomy and Surgery, Chapel Street, Grosvenor Square.
Spry, Mr. Berlin (German Correspondent)
Steel, J. M.D. Marchmont-street, Burton Crescent.
Vickers, Mr. Thayer-street, Manchester Square.
Ward, Joseph, Esq. Surgeon, Ewell, Surrey.
Wilson, Dr. James R. Fellow of the Royal Coll. of Physicians of London, Rue Richepanse, Paris.
Williamson, Mr. Devonshire Sq. Bishopsgate.
Whittley, Mr. Surg. Swaffham, Norfolk.
Wolfe, Mr. Chester-le-street.

### INDEX.

Abdominal inflammation, case of, 528
Abernethy versus the Lancet.... 297
Abortion, the powers of medicines ..................... 418
Abscess of the lungs .......... 511
Accoucheurs, unjust aspersions on 175
Account of the Penitentiary Endemic ....................... 1
Acetate of lead, its properties .. 162
Acetate of lead, its antidote.... 163
Acetate of lead, is it to be used? 355
Acoustic Surgery, Mr. Buchanan's illustrations of ............. 111
Acupuncture, culpable neglect in 300
Acupuncture, M. Pelletan on ... 257
Acupuncture, Mr. Bally's trials of 260
Acupuncturation, Dr. Webster on 562
Allan, Mr. his contrivance for perforated soft palate........ 548
Aloes, Mr. Brande's account of, 145
Alum, as a styptic............... 157
Amputation at the hip, Walther's case ...................... 551
Amputation, partial, of the hand, case of...................... 267
Analecta Minora.............. 587
Analogy between plague and yellow fever .................. 66
Analogy between the effects of poison on man and brutes.... 184
Analysis of medical evidence, Dr. Smith's .................... 169
Anatomie Vivante.............. 600
Aneurism of the heart, depletion in ........................... 101
Aneurism of the heart in young people ....................... 241
Angina œdematosa, cases of.... 205
Animal effluvia, their infectious powers .................... 67
Anomalies in eruptions ........ 445
Anti-contagionists, a *damper* for them ...................... 292
Antidotes...................... 589
Apothecaries, report of........ 599
Appendix to Parry's Elements of Pathology .................. 188
Arsenic mixed with dumplings, Fenning's case.............. 179
Arsenic does *not* prevent the *rising* of dough ............. 181
Arsenic, case of poisoning by.... 289
Arsenic does *not* blacken knives 182
Arterial elasticity and tonicity .. 196
Artificial nose, Mr. Snell's...... 305
Artificial aid in parturition.... 408
Associated apothecaries, their annual report ................ 309
Atmosphere, its influence in cholera.......................... 135
Auscultation in pneumonia ... 513
Average of forceps cases in midwifery...................... 399
Bad health, a cause of distortion 370
Baker, Dr. his case of disease of the heart .................. 529
Bandage in large abdominal wounds ...................... 338
Bandagings and plaster in incised wounds .................... 546
Barley water poisoned by arsenic 290
Barry, Dr. his ideas on the circulation.................... 314
Belcher, Dr. his case of purpura hæmorrhagica .. ........... 284
Bell, Mr. his lectures against Sir A Cooper, on the thigh-bone, &c. 41
Belladonna in tic douloureux .. 300
Bellard, M. his cases of paralysis of the portio dura............ 204
Bibliographical Record ........ 301
Bidois, Dr. his treatment of pericarditis ..................... 242
Biliary ducts, obliteration of the 237
Bistoury and knife in extirpation of the tonsils................ 545
Blandy, Miss, her trial.......... 179
Bleeding, in dissection wounds, Mr. Shaw on................ 253
Bleeding, in dissection wounds, Dr. Thomson on............ 256
Bleeding, in difficult parturition from rigidity............... 420
Bleeding in insanity............ 110
Bleeding, *ad deliquium*, in laryngitis......................... 207
Bleeding, its effects in a severe case of laryngitis............ 210
Blood-letting in tetanus suggested 395
Blood-letting, in cholera morbus 138
Bond, Mr. his case of rupture of the perineum .............. 288
Bouillaud, Dr. his cases of angina œdematosa ................ 205
Bowels, complicated affections of the........................ 7
Brande, Mr. his Manual of Pharmacy ..................... 141
Brandreth, Dr. his case of hydrophobia ..................... 534
Brandreth, Dr. his case of hydrophobia...................... 575

Bronchia, cases of dilatation of its tubes.................... 501
Bronchial inflammation and its consequences................. 504
Bronchial disease, new kind of.. 526
Bronchitis, account of........... 496
Bronchitis, sputa in.............. 506
Broussais' doctrine, proofs of it 325
Broussaiism and Brunonianism estimated ..................... 334
Buboes, treated by iodine frictions ....................... 277
Buboes, syphilitic and otherwise 478
Buchanan, M. on acoustics...... 111
Buckthorn, is it useful?........ 161
Cæsarean section, Mr. Ruth's case of ..................... 285
Cæsarean section, culpable adoption of ..................... 286
Calculus in the colon ........... 532
Calderini, Dr. his opinion of euphorbia lathyrus ............. 273
Calomel in Indian diseases...... 576
Calomel in cholera.............. 137
Calomel, scruple doses of, in dysentery ..................... 271
Calomel, large doses of, in meningitis ...................... 280
Canning, Mr. his speech on contagion...................... 294
Carmichael Mr. on the venereal.. 435
Carotid artery wounded, curious case of .................... 244
Case of dropsy, cured by depletion........................ 89
Cataplasms used in the French Hospitals .................... 351
Cathartine in senna leaves .... 158
Caution necessary in using the forceps ..................... 432
Cellular inflammation, Dr. Duncan on ..................... 212
Cephalitis simulating hepatitis.. 216
Cerumenous glands, defective action of ..................... 114
Chancre and gonorrhœa considered ..................... 448
Chancre, definition of........... 477
Change of air, its beneficial effect 30
Chest, cases of penetrating wounds of the .............. 314
Cholera, Mr. Scot's report of.... 121
Chorea, various authors on...... 563
Chilblains, a "morceau" for.... 353
Children, Dr. Kennedy on the management of............... 485
Civilized and savage life contrasted ..................... 190
Classification of venereal diseases 443
Climates, have they changed? .. 318
Clinique Medicale, by M. Andral 493
Clitoris, extirpation of.......... 558
Cloathing of infants, cautions respecting.................... 489
Colchicum, gathering of the flowers ......................... 573
Cold water, affusions of, in derangement................... 110
Cold affusion in poisoning by laudanum ..................... 276
Colica pictonum, its treatment by "les Pères de la Charité" .. 364
Colica pictonum, leeches in.... 528
Collyrium, acetate of lead a.... 163
Compound fractures, Larrey's treatment of ............... 269
Concussion of the spine, Mr. Dundas's case of............ 225
Confinement in distortion, injurious ..................... 374
Consecutive dislocation of the femur ..................... 55
Constitutional syphilitic symptoms ...................... 479
Constitutional irritation from injuries ...................... 388
Consumption, position in ...... 497
Contagion, arguments of its opponents ...................... 67
Contagion, believed in by very ancient authors................ 578
Contracted joints, shampooing in 382
Contracted vagina, remarkable case of ..................... 420
Contraction of muscles in curved spine ...................... 369
Convulsions during labour...... 427
Copaiba, balsam of, its properties 151
Copaiba in gonorrhœa, formula for it ...................... 152
Coroner's inquest at St. George's 583
Corpora lutea, found in a child 5 years old.................... 588
Costal abscess, communicating with the bronchiæ .......... 213
Coventry, Dr. his remarks on the water of the Mohawk valley, and its influence on goitre.... 229
Cradles, their often dangerous effects ...................... 490
Croup, Dr. Jadelot's treatment of 348
Cruelty, physiological, to animals 198
Cullerier, M. his treatment of syphilis ..................... 105
Curious case of dyspnœa........ 219
Cystitis hepatica, fatal instance of 222
Davies, Mr. his case of phlegmasia dolens ................ 540
Davis, Dr. his elements of midwifery...................... 396

Davis, Dr. his improved forceps.. 408
Davis, Dr. on the forceps ....... 598
Deafness in old people......... 299
Death, from epileptiform attacks 218
Defect of power in arteries, a cause of predisposition ...... 197
Definitions of contagion and infection .................... 68
Delirium during labour, bleeding in 423
Diaphragm, its action prevented by stays.................. 192
Diastasis of the vertebræ, case of 44
Diastasis of the epiphysis of the femur.................... 49
Diet, its effect on the Millbank prisoners ................. 4
Differences between yellow and remittent fever ............. 77
Digestive organs in, and prior to, scrophula ................. 483
Dilatation of the bronchial tubes 500
Dilations of the bronchial tubes 217
Disease of the heart, Dr. Baker's case of .................. 529
Dislocation of the vertebræ in curvature .................. 376
Dissection wounds, Mr. Shaw on 249
Dissection wounds, Dr. Thomson's own case............... 253
Dissection wounds, their effects on the system .............. 254
Dissection wounds, American practice in.................. 300
Dissection, very able, of a hydrophobic patient............. 534
Distortion, does it begin in the loins or back ?............. 368
Disturbance of speech, curious case of .................. 526
Domesticated animals, their maladies .................. 195
Donellan's case, Mr. Hunter's evidence in ................ 183
Donnall's trial for poisoning Mrs. Downing ................. 183
Doses, large, of tartarised antimony ......... ......... 106
Dough *rises* when arsenic is mixed with it .................. 181
Dropsy, Dr. Venables on........ 88
Dropsy (encysted) of the liver, case of .................. 224
Drunkards, state of the stomach in ................... .... 531
Dumeril, M. his mystical collar.. 358
Duncan, Dr. his case of cellular inflammation ............... 212
Dupuytren, M. his tonic potion.. 356
Dupuytren, M. his powder for gangrene .................. 357
Dupuytren, M. his merits........ 363
Dysentery, P. Recamier's ideas of 220
Dysentery in the Fly sloop of war 270
Eau bénite, formula for it...... 356
Edinburgh Senate, their regulations ...................... 294
Education of girls, its evils..... 372
Education, its effects upon the human frame ............... 190
Effects of opium, taken in quantity .................... 155
Egophony and resonance of voice 515
Emanations from marshes, queries as to their nature........ 320
Emetics, moxa, and blisters in insanity.................. 110
Emphysema from the violence of a labour.................. 419
Enlargement of the heart, case of 536
Entologists *v.* the Broussaians.. 326
Epilepsy, nitrate of silver in..... 160
Epilepsy, musk and zinc in...... 167
Epilepsy, its 'local habitation' in the cerebellum............. 223
Ergot of rye, remarks on........ 418
Eruption, papular, history of.... 449
Eruptions, difficulties in distinguishing them .............. 462
Erysipelas, ol. terebinth. in...... 576
Esquirol, M. his treatment of insanity.................... 107
Etiology of the Penitentiary endemic.................... 28
Etiology of epidemics, Dr. Smith's 62
Euphorbia lathyrus, a substitute for croton................. 273
Euphorbia lathyrus ............ 589
Exercise in distortion.......... 380
Exhaustion, in parturition, Dr. Davis on ................ 410
Expectoration in pneumonia.... 516
Extirpation of the tonsils, Mr. Pettigrew's ................. 545
Extirpation of the uterus, Dr. Siebold's case of............... 264
Extirpation of the uterus, Dr. Holscher's case of............... 266
Extraction of the ovaria considered .................... 336
Extraneous bodies in the ear, extraction of................. 113
Fasciculus of engravings from Chatham ........... ........ 57
Fatal case of fever............. 24
Fatigue, a plea against exercise.. 193
Fatty matters in the stomach taken for arsenic............. 290
Fenning, E. her trial.......... 179
Fever, idiopathic, cases of...... 525
Fevers, intermittent and remittent 75

Fomentations, some choice ones 351
Forcible stretching of the spine, Mr. Shaw on. 375
Forceps, its history. 407
Forceps, when to be used. 409
Forceps, average estimate of its use 398
Forceps, its dangers and evils pointed out 428
Forceps, its employment considered 403
Formulary of the French hospital medicines 350
Fouquier, M. his views 99
Francis, Dr. his case of laryngitis 209
Fractured spine, symptoms of. 43
Fractures of the patella, Amesbury's apparatus 301
French 'amour propre' instanced 349
Frost, Mr. on the gathering of the colchicum flowers 573
Fumigation, mercurial, Dr. Gibson on. 568
Fungus hæmatodes, case of. 227
Fungoid tumour of the uterus, case of 287
Gallic *practice* in cephalitis 216
Ganglia of the sympathetic, state in tetanus 388
Ganglion, Dr. Cumin's mode of treating 551
Gangrene after extraction of an ovarium 342
Gargle, a smart mercurial one. 352
Gastritis, its prevalence in phthisical patients 530
Gastroenteritis, how it causes death 330
Gibson, Dr. on mercurial fumigation 568
Goitre, its etiology 228
Gonorrhœa, copaiba in 152
Gonorrhœa, treatment of 456
Gonorrhœa, case of, treated by iodine 279
Guthrie, Mr. and Professor Walther 553
Guthrie, Mr. his mode of amputating at the hip 552
Habits, their influence on the constitution 195
Hæmatemesis, quickly fatal case of 240
Half-blindness, curious state of. 201
Halford, Sir H. his oration 586
Health, how influenced by education, &c. 191
Heiniker, Dr. his complaint answered 600
Hemicrania, cured by acupuncture 263
Hepatization, red, case of 518
Hepatization, objections to the term 509
Hereditary nature of diseases. 187
Hernia humoralis, cases of, cured by iodine 278
Herpes genitalis 556
Hip-joint amputation, objections to 49
Histoire des Marais, M. Monfalcon's 317
Holscher, Dr. his extirpation of the uterus 266
Hôpital de la Charité 98
Hôpital des Enfans, account of it 344
Horse-exercise, Dr. Parry's opinion of 193
Hospital Reports, Mr. Recamier's 217
Hospital Reports, from l'Hotel Dieu 524
Hospital surgeons, in England 584
Hôtel Dieu, sketch of it 96
Howison, Dr. his case of enlarged heart 536
Hunter, Mr. his evidence on Donellan's trial 183
Hunter, Dr. his influence on midwifery 402
Hydro-cyanic acid, its dose and strength 149
Hydrocephalus, drawing off the water in 306
Hydrocephalus acutus, Dr. Jadelot's treatment of 348
Hydrocephalus, Dr. H. Davies on 571
Hydrophobia, Dr. Booth on 571
Hydrophobia, Dr. Brandreth's case of 575
Hydrophobia, state of the nerves in 534
Hydrophobia, prevented by the actual cautery 299
Hysteria, its Protean forms 385
Idiopathic fever, cases of 525
Idiopathic tetanus, cases of 391
Idiotism, remarkable case of, cured 557
Iliac tumour in parturition, case of 416
Importance of accoucheurs 175
*Important* works on the venereal 436
Improvements on the forceps, Dr. Davis's 433
Incised wounds, Mr. Symes' treatment 545
Inclined plane, its advantages considered 373
Indian diseases, calomel in 576
Infants, newly born, when they should be put to the breast 488

Infection, remarks on.......... 83
Infiltration of urine, in lithotomy, prevented.................. 248
Inflammation and fever not identical........................ 88
Inflammation of the retina, anglicè, iritis.................. 362
Inflammation of the lungs, its progress...................... 510
Inflammation and reddening considered...................... 496
Inflammation, tracheal and laryngeal................... 347
Influenza, surmise on a cause of it 449
Injection of the meatus auditorius 112
Injections employed in France.. 352
Injury to the spinal column.... 225
Inquests, abuses of............ 173
Insanity, not always elucidated by dissection............... 231
Instruments, parturient, Dr. Davis's........................ 406
Instruments in twin-births...... 417
Instruments, when indicated in labour.................... 424
Intelligence, &c............... 313
Intermittent affection of the chest, case of.................... 272
Intermittents, a stumbling-block to the Broussaians.......... 328
Intermittents, Broussais' views of 332
Intermittents, sulph. zinci in.. 168
Intestinal calculi, Mr. Torbet's case of...................... 532
Intestinal calculi, causes of.... 533
Introduction of the forceps...... 429
Intus-susception, operation for.. 537
Intus-susception, interesting case of......................... 539
Iodine in venereal affections.... 277
Iodine in sulphureous springs.. 298
Iritis, account of............. 451
Iritis, mercury and antiphlogistics in...................... 460
Iron nail swallowed............ 301
Irritable fever, fatal case of ... 211
Itch, the best remedies for..... 570
Jackson, Dr. his case of paracentesis thoracis............... 542
Jadelot, Dr. his *pratique*........ 345
Joints, their injuries from exercise 381
Jones, Mr. his remarks on dysentery........................ 272
Jury, its powers on medical investigations................... 585
Kennedy, Dr. on the management of children............ 485
Kennedy, Dr. his case of intestinal concretion .............. 533
Knives *not* blackened by arsenic 182
Labour, case of cessation of.... 414
Laryngitis, extensive bleedings in 207
Laryngitis, Dr. Francis's case of.. 209
Laryngitis, Dr. Johnson's case of 210
Larynx, extraordinary affection of its muscles............... 385
Larynx, ulceration of it........ 468
Larynx, phagedenic ulceration of 472
Larynx, diseased, tracheotomy for 550
Late hours, ill effects of......... 195
Lateral curvature, Mr. Shaw on.. 366
Latham, Dr. his work on the Penitentiary endemic............ 1
Laudanum, as an application in dissection wounds ............ 250
Laudanum extracted from the stomach...................... 276
Laurel water, its effects upon dogs 186
Learned men bad practitioners.. 166
Leeches, nostrum for making them *take*..................... 587
Lerminier, Dr. his doctrines.... 101
Ligatures in abdominal wounds 338
Liniments and lavements....... 353
Lithotomy, Mr. Averill's operation 539
Lithotomy, Mr. Liston's remarks on 247
Lizars, Mr. on diseased ovaria.. 335
Lungs, case of inflammation of the 517
Machines for pressing supposed dislocated vertebræ........... 376
Man's appearance in marshy countries ................ 321
Manual of pharmacy, Mr. Brande's .................... 141
Marshall Mr. his evidence on Fenning's trial.................. 180
Marx and Omodei, *v.* Dr. Maclean 578
Matter, forming after dissection wounds .................... 250
Meatus, purulent discharge from the ......................... 118
Medical witnesses, their perplexities....................... 172
Medical liberality, anecdote of.. 178
Medicine, not a trade.......... 177
Meningitis, Mr. Davies's case of 279
Mercurial fumigation, Dr. Gibson on ....................... 568
Mercurial ointment, new mode of preparing .................. 588
Mercury, its action on the system 438
Mercury and its opponents...... 440
Mercury in the papular venereal 451
Mercury in the phagedenic disease 469
Mercury in syphilis ............ 481
Mercury, as a *preventive* of hydrophobia ................... 536
Mercury, its efficacy at Millbank 12
Meteoration, epidemic.......... 85
Miasm from animal decomposition 69

Miasm, distinctions of.......... 70
Miasm defined ................ 319
Midwifery, when first practised by a surgeon .................. 401
Midwifery, is it to be despised?.. 404
Monfalcon, M. his Histoire des Marais ...................... 317
Moral causes, their effects upon the heart .................. 242
Morbid appearances after death, from dysentery.............. 9
Mortality of children, averaged in London.................. 483
Mortality in pulmonic inflammation...................... 527
Muscles, can they produce distortion...................... 368
Muscles, nervous contraction of.. 384
Muscularity of the urethra...... 164
Muscular power, curious instance of...................... 380
Needles, great number extracted from the body............. 559
Nerves of motion and sensation, their sizes................ 226
Nervous disorders combined with dysentery .................. 16
Nervous disorders cured by mercury .................... 20
Neuralgiæ, cases of, cured by acupuncture ................ 258
Neuralgiæ, cases of, treated by do. 262
Neuritis, in a hydrophobic patient 534
Nicholls, Dr. his case of tetanus 283
Nitrate of silver, history and properties of ................ 160
Nitrate of silver, antidote to.... 161
Nitre in menorrhagia.......... 298
Nodes, unfrequent in the papular venereal.................. 452
Nodes in phagedena.......... 469
Nodes, treatment of............ 475
Obligation of the practitioner to attend inquests.............. 171
Obliteration of the bile-ducts, cases of.................. 237
Oil of bitter almonds, its nature &c 148
Ointments, mercurial and iodidic 353
Old people, on their diseases.... 571
Opacities of the cornea, their treatment .................. 359
Operations, bloody, remarks on.. 267
Opium, its efficacy in cholera.... 137
Opium, its powers............ 154
Opium, cautions respecting its employment ................ 156
Opium, too much dreaded in inflammations ................ 223
Optic nerves of the simia aygula 203
Organic disease, what is it ?.... 92
Ovaria, extraction of, considered 344
Ovaria, diseased, Mr. Lizars on 335
Ovaria, case of extraction of.... 337
Ovarian dropsy, old operations for 588
Ovarium, extirpated, case of.... 341
Ovarium, attempted extraction of 342
Papular venereal disease, account of........................ 447
Papular eruption and mercury.. 458
Par vagum, M. Breschet's memoir 200
Paracentesis thoracis, Dr. Jackson's case .................. 542
Paracentesis thoracis, successful case of .................... 219
Paralysis of the portio dura...... 203
Paralysis of the portio dura, Dr. Delafield's cases ............ 281
Paralysis from a fall, case of.... 58
Parisian hospitals, Dr. Ratier on the 94
Parry, Dr. his Elements of Pathology, &c................ 188
Parturition, exhaustion in, case of 411
Pathological action of marsh effluvium ...................... 322
Pathology, definition of........ 89
Pelvic tumours during parturition 421
Pelvis, deformed, remarks on.... 423
Penetrating wounds of the chest, cases of .................... 314
Penitentiary, Dr. Latham's account of its endemic........ 1
Pères de la Charité, their curious treatment of colica pictonum.. 364
Perforation of the stomach, case of 221
Perforations of the soft palate.. 547
Pericarditis, ill treated case of.. 242
Pericarditis and gastritis, case of 527
Perineum, severe rupture of.... 288
Periodical bleedings, after amputation ...................... 267
Periscope .................... 198
Periscope, preface to it........ 523
Phagedenic venereal disease, description of it ................ 463
Phagedenic ulcer, its constitutional symptoms ............ 466
Phagedenic affections of the joints 468
Phagedenic ulcer Mr. Carmichael's treatment of................ 470
Pharmacy, manual of,—by Mr. Brande .................... 141
Philip, Dr. on galvanic experiments.................... 589
Phlegmasia dolens, Mr. Davies's case of .................... 540
Phlegmasia dolens, M. Velpeau on 233
Phlegmasia dolens, Dr. Davis's case of .................... 541
Phlegmasia dolens, remarks on.. 237
Phthisis pulmonalis and bronchitis 496

Phthisis, state of the stomach in 530
Physicians, opening of their new college 586
Pills, a rather odd mercurial one 354
Plague of Athens, its probable nature 74
Plague and yellow fever, differences between 82
Pneumonia 508
Pneumonia, its site 512
Pneumonia, auscultation in 513
Pneumonia ending in gangrene, case 521
Poisoning, case of, by euphorbia peplus 275
Poisoning by arsenic, Orfila's case 289
Poisoning by arsenic, curious instance of 291
Poisoning, by arsenic and sublimate 565
Pomegranate root, for the expulsion of tape-worm 574
Position of the limb during sawing of the bone 50
Post-mortem appearances, in cholera 131
Pratt, Mr. his prediction 38
Precautions in opening bodies 255
Predisposition, a want of power in arteries 197
Preputii herpes 556
Professor Delpech's case of wounded carotid 244
Proofs of Sir A. Cooper's early opinions of non-union, in fracture of the neck of the femur 53
Properties and history of the euphorbia lathyrus 274
Pulmonic diseases, Dr. Cobb on 217
Purpura hæmorrhagica, Dr. Belcher's case of 284
Pustular venereal disease, character of the 461
Pyroligneous acid, its action on the ear 116
Quackery & the regulars, in France 344
Quarantine, late regulations for 292
Ratier, Dr. on the Parisian hospitals 95, 344
Recamier, M. his Hospital Reports 217
Receipt for an opening aloetic pill 147
Red and grey hepatization of the lungs, case 519
Report, Mr. Scot's, of the Madras cholera 121
Respiration and pulse of children 487
Rete mucosum, blackened by nitrate of silver 161
Retention of urine, tinct. ferri. mur. in 165
Retention and suppression, difference between 165
Rheumatism, chronic and acute, acupuncture in 261
Rigidity of the passage, during labour 419
Ruth, Mr. his curious case of Cæsarean section 285
Rupture of the perineum, case of 287
Sangrado practice revived 299
Scaly eruptions, mercury in 473
Scirrhous uterus, unhappy case of 414
Scot, Mr. his report of cholera 121
Scott, Dr. on puncturing the brain in hydrocephalus 306
Scott, Dr. his case of cystitis hepatica 222
Scrophula, M. Dupuytren's practice in 358
Scrophula, state of the digestive organs in 483
Scrophula, Mr. Rennie on 565
Scrotal tumour, curious instance of 227
Scurvy, its appearance and symptoms among the Millbank prisoners 6
Sea-water-baths to be established 601
Secrecy, in the treatment of female complaints 176
Semi-decussation of the optic nerves 201
Senna leaves, account of 157
Shampooing and rubbing considered 382
Shaw, Mr. on dissection wounds 249
Shaw, Mr. on lateral curvature, &c. 366
Shoulders, in incipient curvature 367
Siebold, Dr. his case of extirpated uterus 264
Sloughing ulcer, its progress and origin 465
Sloughing ulcer and chancre, their distinctions 478
Smith, Dr. his arrangement of miasms 71
Smith, Dr. his work on Medical Evidence 169
Smith, Dr. Gordon, his reclamation 596
Softening of the lung, grey, case of 520
South America, medical science in 313
Spasmodic pains in the joints 386
Spirits, effects of, upon animals 184
Spontaneous salivation in syphilis 480
Spurzheim, Dr. his view of insanity 232
Sputa of bronchitis 506
St. George's Hospital, inquest at 583
State of the blood in cholera 129
Stays injurious, and yet useful, in distortion 377
Stethoscope, testimony to its value 495

Stoop, erroneous modes of treating it ........................ 379
Subluxation of the vertebræ, characters of ........................ 44
Suckling infants, duties of the mother........................ 488
Sulphate of zinc, its powers.... 169
Sulphate of quinine, from residuous bark ........................ 574
Supposed union of fractured *cervix femoris* ........................ 61
Suppression of urine, on healing an old ulcer........................ 240
Surgeons of hospitals, hints to.. 524
Swan, Mr. on tetanus.......... 387
Symes, Mr. his treatment of incised wounds........................ 545
Syphilis treated by Van Swieten's liquor........................ 104
Syphilis, mercury in............ 360
Syphilis and its relatives...... 437
Syphilitic and syphiloid pains.... 481
Tape-worm, expelled by the pomegranate root ........................ 574
Tartar emetic, large doses of, in pneumonia ........................ 573
Tetanus, Mr. Swan on........... 387
Tetanus, stimulants in........... 283
Tetters (phagedenic) new treatment of ........................ 360
Thickening of the bronchial membrane ........................ 499
Thigh-bone, necrosis of ........ 48
Thigh-bone, supposed fracture of its neck ........................ 389
Thomson, Dr. his case of dissection wound........................ 253
Throat, phagedenic ulcers of this part........................ 473
Tinctura ferri muriatis, as a tonic 163
Tinctura ferri muriatis, in retention of urine ........................ 164
Tisane de Feltz, its composition 357
Tonic treatment of dissection wounds ........................ 251
Tracheotomy in laryngeal ulceration........................ 475
Tracheotomy, Mr. Goodeve's case of 549
Transplantation of a tooth, dreadful consequences from........ 487
Traumatic tetanus, state of the sympathetic in............... 393
Treatment of tetanus........... 395
Treatment of dysentery pursued at Millbank ................ 11
Trephining the spine, Mr. Bell's criticisms on ................ 46
Tumours, hæmorrhagic, Velpeau 226
Turning in parturition, Dr. Davis's ideas on................ 426
Twin-births, instruments in.... 417
Typhus, its causes ............ 82
Ulceration of the throat, in the phagedenic disease .......... 467
Ulceration of the nostrils, symptoms of ........................ 468
Ulceration of the throat, syphilitic 480
Ulceration of the trachea...... 499
Ulcers, primary venereal, their treatment ........................ 453
Urethra, sympathetic irritation of 549
Uterine tumour, Mr. Lightfoot's case of........................ 286
Uterus extirpated, dreadful operation........................ 265
Van Swieten's liquor, recipe for 101
Vapour bath, a cheap and effective one........................ 383
Variolo-vaccination, Dr. Ferguson's proposal ................ 295
Variolous eruption mitigated.... 589
Veins inflamed, in phlegmasia dolens, fancied cases of......... 234
Velpeau, M. his paper on certain tumours........................ 226
Venables, Dr. on dropsy.......... 88
Venereal diseases, Mr. Carmichael 435
Venereal poisons, their plurality argued ........................ 441
Venesection, &c. in wounds of large arteries................ 244
Vesication, difficulty of, in cholera 127
Vitiated secretions, denied by Broussais ........................ 331
Vivisections, remarks on......... 198
Walcheren fever, Monfalcon's remarks on ........................ 323
Walther, Dr. his amputation at the hip ........................ 551
Want of exercise predisposes to disease ........................ 192
Warm climates, rarity of distortion in........................ 371
Warts, venereal, acetic acid for.. 455
Water, its influence in causing goitre........................ 228
Webster, Dr. on acupuncturation 562
Whiskey, an injection instead of salt........................ 300
White, Mr. his case of sympathetic irritation of the urethra...... 549
Wine, its too frequent use...... 194
Wollaston, Dr. on the optic nerves 201
Wood, Mr. on tartar emetic in pneumonia........................ 573
Woodville's experiments on vaccination........................ 296
Yaws, remarks on ............ 439
Yellow fever, Dr. Smith's views of 78
Yellow fever, Dr. Belcher on.... 568

